# The iGreenGO Study: The Clinical Role of Indocyanine Green Imaging Fluorescence in Modifying the Surgeon’s Conduct During the Surgical Treatment of Advanced Gastric Cancer—Study Protocol for an International Multicenter Prospective Study

**DOI:** 10.3389/fonc.2022.854754

**Published:** 2022-03-17

**Authors:** Pietro Maria Lombardi, Michele Mazzola, Vincenzo Nicastro, Simone Giacopuzzi, Gian Luca Baiocchi, Carlo Castoro, Riccardo Rosati, Uberto Fumagalli Romario, Luigi Bonavina, Fabio Staderini, Ines Gockel, Dario Gregori, Paolo De Martini, Monica Gualtierotti, Maria Danieli, Simona Beretta, Massimiliano Mutignani, Edoardo Forti, Giovanni Ferrari

**Affiliations:** ^1^ Unit of Foregut Surgery, IRCCS Humanitas Research Hospital, Milan, Italy; ^2^ Division of Minimally-Invasive Surgical Oncology, Niguarda Cancer Center, ASST Grande Ospedale Metropolitano Niguarda, Milan, Italy; ^3^ General and Upper GI Surgery Division, University of Verona, Verona, Italy; ^4^ General Surgery, ASST Cremona, Cremona, Italy; ^5^ Department of Clinical and Experimental Sciences, University of Brescia, Brescia, Italy; ^6^ Department of Biomedical Sciences, Humanitas University, Milan, Italy; ^7^ Department of Gastrointestinal Surgery, San Raffaele Hospital IRCCS, Faculty of Medicine and Surgery, Vita-Salute San Raffaele University, Milan, Italy; ^8^ Department of Digestive Surgery, Istituto Europeo di Oncologia IRCCS, Milan, Italy; ^9^ Division of General and Foregut Surgery, Department of Biomedical Sciences for Health, Policlinico San Donato, University of Milan, Milan, Italy; ^10^ Digestive Surgery Unit, Department of Clinical and Experimental Medicine, Careggi University Hospital of Florence, Florence, Italy; ^11^ Department of Visceral, Transplantation, Thoracic and Vascular Surgery, University Hospital Leipzig, Leipzig, Germany; ^12^ Unit of Biostatistics, Epidemiology and Public Health, Department of Cardiac Thoracic Vascular Sciences and Public Health, University of Padova, Padova, Italy; ^13^ Data Management Unit, Division of Minimally-Invasive Surgical Oncology, Niguarda Cancer Center, ASST Grande Ospedale Metropolitano Niguarda, Milan, Italy; ^14^ Department of Digestive Endoscopy, Niguarda Cancer Center, ASST Grande Ospedale Metropolitano Niguarda, Milan, Italy

**Keywords:** gastric cancer, advanced, indocyanine green (ICG), surgery, D2 lymphadenectomy, surgical conduct

## Abstract

**Background:**

The near-infrared/indocyanine green imaging fluorescence (NIR/ICG) technology is showing promising results in several fields of surgical oncology. The clinical value of NIR/ICG technology in the surgical treatment of advanced gastric cancer (AGC) is not clearly established.

**Methods:**

This is the protocol of the “iGreenGO” (indocyanine Green Gastric Observation) Study, a national prospective multicenter study. Western patients who undergo curative-intent gastrectomy with D2 lymphadenectomy for AGC constitute the study cohort. All the patients undergo preoperative upper gastrointestinal endoscopy for submucosal peritumoral ICG injection at the most 20 h before surgery. Intraoperative endoscopic injection before starting surgical dissection is also allowed. The primary endpoint is the “change in the surgical conduct” (CSC), i.e., the need to perform further dissection after intraoperative NIR/ICG technology activation at the end of D2 lymphadenectomy. Secondary endpoints include the pattern of abdominal fluorescence distribution according to tumor and patient characteristics, the preoperative clinical variables potentially associated with CSC, and the incidence of stage migration due to NIR/ICG application.

**Discussion:**

The iGreenGO Study is the first study to investigate the clinical role of NIR/ICG technology for the surgical treatment of AGC in a large cohort of Western patients. Results from the present study can further clarify the role of NIR/ICG technology in surgical lymphadenectomy for AGC.

## Background

Gastric cancer accounts for 5.7% of all neoplasms in both sexes. It is the fifth most common and the third most lethal tumor in Europe, with a 5-year overall survival (OS) rate of 25% (1, 2).

Although several (neo)adjuvant treatments have been developed to improve survival outcomes (3), surgery remains the mainstay of treatment of resectable gastric cancer and the only curative option (4).

Surgical intervention was traditionally performed *via* laparotomy; however, the minimally invasive approach has gained attention over the last decade, demonstrating better short-term outcomes (e.g., lower blood losses, reduced postoperative pain, and reduced hospital stay) (5, 6) and comparable long-term oncological outcomes (7) as compared with the open approach.

Regardless of the surgical approach, margin-negative resection and adequate lymphadenectomy are the major treatment-related prognostic factors (4).

In patients with resectable advanced gastric cancer (AGC), the Japanese Gastric Cancer Association (JGCA) guidelines (4, 8) recommend D2 lymphadenectomy, consisting in the removal of the lymph nodes (LNs) along the left gastric artery, the common hepatic, the splenic artery, and the coeliac axis, in addition to the perigastric LNs (9). In carefully selected patients, a nodal dissection beyond the D2, the so-called D2+ lymphadenectomy, is recommended (9).

Since nodal involvement is one of the most important prognostic factors for OS and disease-free survival (DFS) (10, 11), several efforts have been made to help the surgeon to optimize lymphadenectomy. Among these, the application of indocyanine green (ICG) together with light at wavelengths in the near-infrared range (NIR) is showing interesting results.

ICG is a fluorescent cyanine dye approved for medical uses by the FDA in the late 1950s. Administered intravenously or through submucosal visceral injection, it allows the visualization of anatomical structures (12). Injected in any tissue (submucosal visceral, intramammary, intracutaneous, etc.), ICG binds to interstitial proteins (albumin, globulins, etc.) and enters the lymphatic vessels of the injected site. So, fluo-labeled “proteins” are taken up by the reticuloendothelial cells in the lymph nodes on the lymphatic draining pathways. The absorption and fluorescence spectrum of ICG is in the NIR region (wavelength 800–1,000 nm) and can be visualized by dedicated infrared-based cameras. ICG has low autofluorescence and high tissue penetration by the wavelengths of the penetrating laser (which excites the ICG), providing better contrast in real-time structure visualization during surgical intervention; for these reasons, over the last years, its use has been preferred over other fluorescent contrast agents, such as methylene blue and fluorescein, which have lower wavelengths (700 and 520 nm, respectively) and therefore suboptimal characteristics for surgical visualization (13).

NIR fluorescence imaging with ICG (defined as “NIR/ICG technology” hereinafter) has been introduced in many surgical fields (14): in colorectal surgery, it is used for the evaluation of real-time visceral perfusion with the aim to reduce the rate of anastomotic leaks (15); in hepatobiliary surgery, it allows the visualization of the biliary structures and the evaluation of liver function (16); and in melanoma and breast cancer surgery, it might be used for sentinel LN detection (17, 18).

In the field of gastric cancer surgery, the NIR/ICG technology was firstly applied in Eastern countries for sentinel LN detection in patients with early gastric cancer (EGC) (19). In Western countries, where the different tumor biology and the absence of a screening program lead to a higher rate of ACG with nodal involvement at first diagnosis (20), the NIR/ICG technology was rather used for abdominal nodal mapping (21, 22) showing promising results in the assessment of lymphadenectomy thoroughness (23–26). However, all the studies investigating NIR/ICG technology focused on its ability to increase the number of harvested LNs, while no data exist regarding its potential role in changing the surgical conduct at the moment of lymphadenectomy (27) allowing an improvement in lymphadenectomy as compared with lymphadenectomy performed without the use of any technology; in addition, no data exist about the role of this hypothetical lymphadenectomy improvement on tumor staging and the subsequent treatment planning.

## Material and Methods

### Objective

The iGreenGO Study aims to investigate whether the intraoperative application of NIR/ICG technology is associated with a change in the surgical conduct (CSC) during curative-intent gastrectomy with D2 lymphadenectomy in a cohort of Western patients affected by AGC.

The preoperative clinical variables potentially associated with CSC will be also investigated.

### Study Design and Setting

The iGreenGO Study is a single-arm prospective observational multicentric study. National and international academic and non-academic hospitals are invited to participate in the study. The duration of the study will be approximately 4 years (3 years for inclusion, 1 year for data analysis and results publication). The enrolment of patients has started since September 2021.

All procedures will be in accordance with the ethical standards of the responsible committee on human experimentation (institutional and regional) and with the Helsinki Declaration of 1975, as revised in Brazil 2013 (28). The Local Ethical Committee reviewed and approved the protocol (380-09062021). The protocol is registered at *clinicaltrial.gov* (NCT04943484). Study results will be reported according to Strengthening the Reporting of Observational Studies in Epidemiology (STROBE) statements (29).

### Study Population

This study includes all patients affected by advanced resectable (cT2–T4a, N0–3, M0) gastric adenocarcinoma.

### Inclusion Criteria

In order to be eligible to participate in this study, a patient must meet all of the following criteria:

- Age ≥18 years old
- Preoperative histologically proven primary adenocarcinoma of the upper, middle, or lower part of the stomach
- Advanced disease (staged cT2–T4a, N0–3, M0 according to the eighth edition of the AJCC TNM Staging System) at diagnosis, in which curative resection can be safely achieved by distal or total gastrectomy with D2 lymphadenectomy *via* a minimally invasive (robotic or laparoscopic) approach, either as first treatment or after neoadjuvant treatment
- Written informed consent signed to preoperative endoscopy and surgical intervention

### Exclusion Criteria

Patients presenting one or more of the following conditions will not be eligible to participate in the iGreenGO Study:

- Previous abdominal surgery interfering with lymphatic drainage of the stomach, including previous gastrectomy, endoscopic mucosal resection, or endoscopic submucosal dissection
- Conversion to laparotomy during surgical dissection for any reason
- Cancer located at the esophagogastric junction [Siewert I, II, III tumors (30)]
- Patients who are candidates for transthoracic esophagectomy, transhiatal extended gastrectomy, or proximal gastrectomy
- Distant metastases and/or direct invasion of the pancreas, spleen, or other adjacent organs at the preoperative and intraoperative examination
- History of allergy to iodine agents
- Women during pregnancy or breastfeeding

### Sample Size Calculation

The sample size is calculated by considering the estimated incidence of intraoperative change in the surgical conduct when using NIR/ICG technology and targeting the precision of the estimates. Data from the literature indirectly suggest an approximate rate of such an event around 17% (27). Perturbing such assumptions (columns) in a sensitivity analysis, the following scenarios are obtained to get an estimated precision of a certain level (rows) with a confidence level of 1 – alpha = 95%, by using the Wilson approach (31, 32). For precisions between 4% and 5%, the expected range of patients will be between 180 and 400, according to adherence to the initial hypothesis made on the incidence of intraoperative change (**Table 1**). This approach in design captures the uncertainty emerging from the literature and the high variability of papers included in the study.

**Table 1:** Sample size calculation.

Incidence of change in surgical conduct							Precision
**20%**	**19%**	**18%**	**17%**	**16%**	**15%**	**14%**	
6,145	5,911	5,668	5,419	5,163	4,896	4,624	**1%**
2,731	2,627	2,518	2,408	2,294	2,176	2,057	**1.50%**
1,535	1,476	1,417	1,354	1,289	1,223	1,157	**2%**
981	944	906	866	826	782	739	**2.50%**
681	655	629	601	574	543	515	**3%**
501	481	462	442	420	400	379	**3.50%**
382	369	352	337	321	307	288	**4%**
302	291	279	266	253	241	229	**4.50%**
245	234	227	217	207	195	186	**5%**

The scenarios are obtained to get an estimated precision (bold values) of a certain level (rows) with a confidence level of 1 – alpha = 95%.

Computations are performed using the R System and the binomSamSize libraries (33, 34).

### Endpoints

The primary endpoint is the CSC at the moment of intraoperative NIR/ICG technology activation after D2 lymphadenectomy performed with the “naked eye.”

The secondary endpoints are the preoperative clinical variables potentially associated with CSC. The other secondary endpoints are as follows: the pattern of abdominal fluorescence distribution according to tumor and patient characteristics, the number of additional lymph nodes retrieved using NIR/ICG technology, the incidence of stage migration due to NIR/ICG application, and the 90-day morbidity and mortality.

### Statistical Analysis

A biostatistician (DG) will perform the statistical analysis. Continuous variables are described as mean (standard deviation) or median (interquartile range) according to distribution; categorical variables are reported as frequency (percentage). Normal distribution of continuous variables is assessed with the Kolmogorov–Smirnov test. Continuous variables are analyzed using Student’s *t*-test or Mann–Whitney test and categorical variables analyzed using Fisher’s exact test or chi-square test as appropriate. All statistical tests are two-sided and a level of <0.05 is used to indicate statistical significance. CSC will be modeled *via* a generalized linear model in the binomial family. The identification of preoperative variables independently associated with CSC will be performed by stepwise selection and using Akaike information criterion (AIC) as benchmark for inclusion. Goodness of fit will be based on the Somers’ Dxy index, adjusted for optimism in accuracy *via* bootstrap resampling (35). A graphic representation of the patterns of fluorescence distribution in the abdominal regions will be represented. Statistical analysis is conducted in R (R Core Team, 2020) (33).

## Treatment of Patients

### Staging

Diagnostic workup includes an upper GI endoscopy with the description of the tumor location and a biopsy for histology. Clinical staging is assessed through the execution of contrast-enhanced computed tomography (CT) scan. On CT scan, suspected LNs are defined as LNs with short-axis diameter >5 and >8 mm in the case of perigastric and extraperigastric nodes, respectively (36). Endoscopic ultrasound (EUS), F-fluorodeoxyglucose (FDG) positron emission tomography (PET)/CT, and staging laparoscopy can also be performed at the discretion of the attending surgical team/tumor board. Preoperative/pretreatment clinical stage is defined according to the eighth edition of the UICC Staging System (TNM) (10).

### Neoadjuvant Chemotherapy

The indication to neoadjuvant chemotherapy (NACT) is evaluated for every patient by the local multidisciplinary tumor board according to national and international guidelines (4, 36). NACT is defined as any chemotherapy received within 8 weeks before surgery.

### Restaging

Restaging is performed after NACT with total body CT scan performed within 4 weeks after completion of NACT in order to rule out progressive disease (PD) or distant metastasis and confirm surgical indication. In the absence of PD or distant metastasis, curative treatment surgery is performed within 6 weeks after NACT completion.

### Step 1: Endoscopic ICG Injection

**Figure 1:**
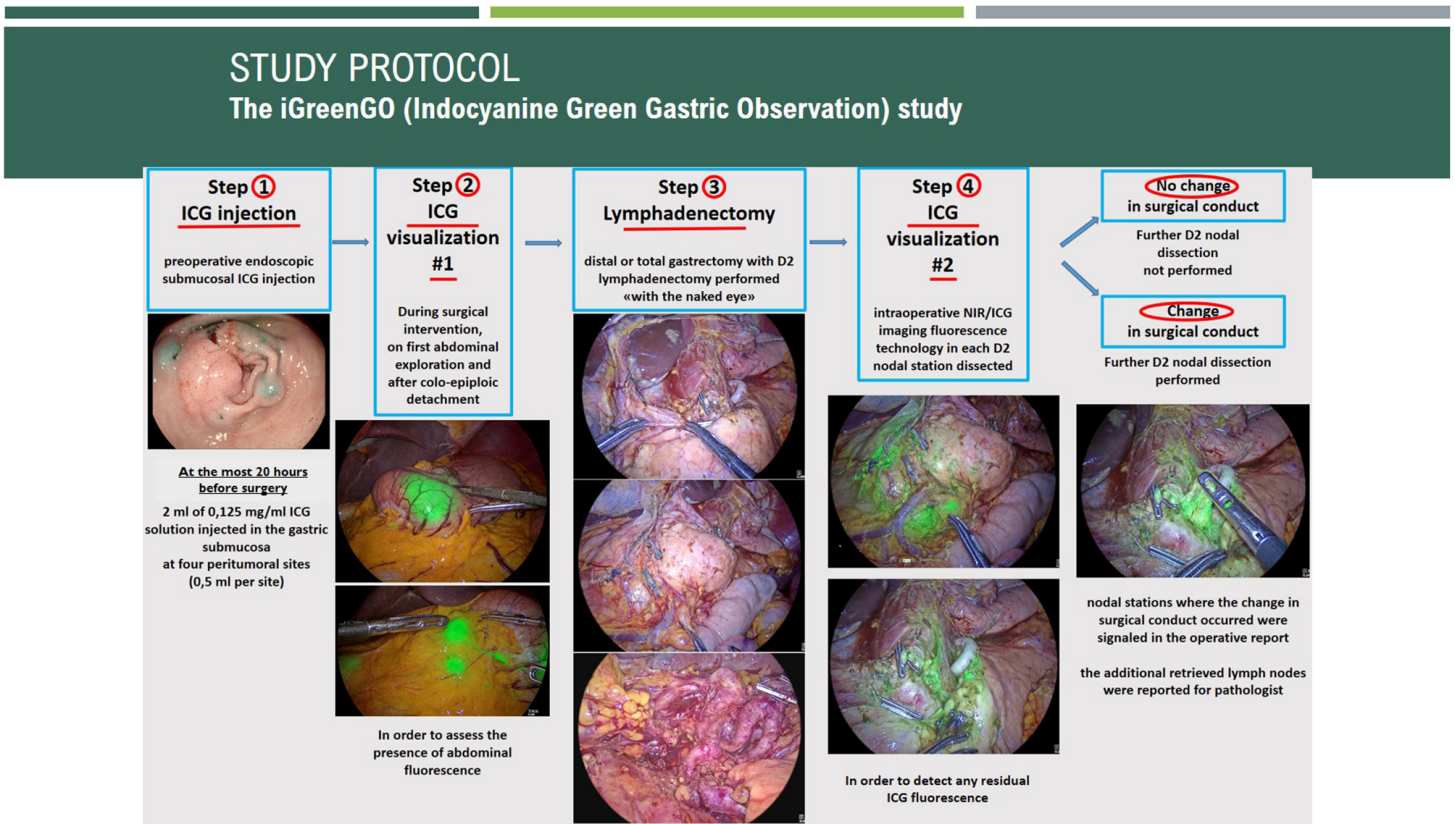
Summary steps of the iGreenGO study. NIR/ICG, near-infrared/indocyanine green.

The steps of the iGreenGO Study are graphically summarized in **Figure 1**.

Patients undergo upper GI endoscopy at any time between 20 h before and the initiation (skin incision) of the planned surgery. The ICG powder (Verdye**^®^** 5 mg/ml, 25 mg powder for solution for injection, Diagnostic Green, Aschheim-Dornach, Germany) is dissolved in sterile water, obtaining a final concentration of 0.125 mg/ml. Two milliliters of the obtained solution is injected in the submucosa with a 23-gauge endoscopic needle (introduced through the flexible scope working channel) into four peritumoral sites, 0.5 ml for each site. In the case of stenotic cancers, ICG injection can be performed at four proximal tumor points.

### Step 2: Intraoperative Application of NIR/ICG Technology (First Time)

Surgical intervention is performed exclusively *via* a minimally invasive approach, either laparoscopic or robotic, at the most 20 h after endoscopic submucosal ICG injection.

Intraoperative application of NIR/ICG technology is accomplished by using special optical devices adapted to fluorescence imaging. Using high-resolution video cameras linked to the ICG surgical endoscope, the technology allows a real-time ICG-enhanced visualization of LNs by toggling seamlessly between white light (which means light in the range of the spectrum visible by the normal human eye, 400–700 nm) and ICG fluorescence mode (NIR wavelength, 800–1,000 nm) in an “overlay way.”

A brief visualization of the operative field with NIR/ICG technology is performed in two moments at the beginning of surgery, during the exploration of the abdominal cavity and after the colo-epiploic detachment, in order to assess the presence of fluorescence in the following perigastric and locoregional sites:

- Lesser gastric curvature (no. 1, 3 nodal stations)
- Greater gastric curvature (no. 2, 4 nodal stations)
- Suprapyloric and hepatoduodenal ligament region (no. 5, 8, 12 nodal stations)
- Gastropancreatic fold (no. 7, 9, 11 nodal stations)
- Duodenopancreatic region (no. 6 nodal stations)
- Tail of the pancreas and splenic hilum region (in the case of total gastrectomy, no. 11 and 10 nodal stations)

ICG-absorbing sites appearing as green spots and emitting fluorescence are defined as fluorescent sites.

### Step 3: Surgical Intervention and Lymphadenectomy

The type of surgical resection is assessed according to the tumor site and is accomplished according to JGCA guidelines (9).

More specifically:

- Distal gastrectomy (DG) with D2 lymphadenectomy includes the resection of two-thirds of the stomach including the pylorus and dissection of 1, 3, 4sb, 4sd, 5, 6, 7, 8a, 9, 11p, and 12a lymph node stations.
- Total gastrectomy (TG) with D2 lymphadenectomy includes the total resection of the stomach including the cardia and pylorus and dissection of 1, 2, 3, 4sa, 4sb, 4d, 5, 6, 7, 8a, 9, 11p, 11d, and 12a lymph node stations.

Digestive tract reconstruction can be accomplished *via* a mini-laparotomy through an abdominal incision not greater than 10 cm. Any abdominal incision greater than 10 cm will be classified as a conversion to open approach, as well as any need to complete the resection phase by any type of laparotomy. A specific sequence of D2 nodal station dissection is not mandatory; the D2 lymphadenectomy technique described by Giacopuzzi et al., which is based on the takeover of specific vascular landmarks, is used in most participating centers (37).

Surgical resection with D2 lymphadenectomy is performed “with the naked eye,” which means using conventional white-light imaging visualization without application of NIR/ICG technology.

### Step 4: Intraoperative Application of NIR/ICG Technology (Second Time) and Definition of CSC

At the end of D2 lymphadenectomy “with the naked eye,” a visualization of the operative field is performed using NIR/ICG technology in order to verify the presence of residual LN in each D2 nodal station.

The CSC is defined in the case of persistence of fluorescence in one or more D2 nodal stations after the completion of D2 lymphadenectomy performed with the “naked eye,” requiring further dissection to remove the residual D2 nodal fluorescent structures.

The nodal stations dissected after NIR/ICG visualization are also recorded.

### Postoperative Management

Postoperative care is accomplished according to the standard practice of each surgical participating institution. Epidural analgesia, early mobilization, and enteral feeding are recommended according to ERAS guidelines (38). Discharge criteria include the absence of fever, effective analgesic oral therapy, mobilization and self-management at least similar to preoperative status, and oral and/or enteral nutrition able to provide at least 60% of the daily energy requirement.

### Pathology

Surgical specimens are processed and analyzed by the pathology department of each participating institution following national and international guidelines (4).

The tissue sample is routinely fixed in 10% neutral formalin and embedded in paraffin. Commercially available monoclonal antibodies such as immunohistochemical stains for CK AE1/AE3 (Dako Omnis^®^) are performed where appropriate. One dedicated pathologist per institution examines the relative resected specimens. Pathologic reports are drawn up reporting morphological subtypes according to Lauren (39) and the WHO Classification of Tumors (40). In the case of NACT, histopathological tumor regression grade is assessed according to Becker’s grading system (41). Further histopathological variables include tumor size, Borrmann classification (42), grade of differentiation, margin status, presence of lymphovascular (LVI) and perineural (PNI) invasion, and lymph node ratio (LNR), expressed as the ratio between positive LNs and the total number of retrieved LNs.

In microscopic vision, an LN is defined as a delimited area of lymphoid cells containing a follicular architecture, with or without a subcapsular sinus. Tumoral cells containing LNs are defined as metastatic. The additional LNs retrieved while using the NIR/ICG technology are signaled to the pathologist by the operating surgeons. Surgical specimens are assessed in order to obtain a final pathologic report, expressed as pTNM/ypTNM, according to the eighth edition of the AJCC Staging System (10).

### Follow-Up

Postoperative follow-up and long-term surveillance are achieved through physical examination, blood test analysis, esophagogastroduodenoscopy (EGDS), and CT scan in accordance with national and international recommendations (43, 44).

### Surgical Quality Control

Participating centers are academic and non-academic hospitals with a history of at least 20 gastrectomies performed *via* a minimally invasive approach. Photographs of the surgical field are taken at the end of nodal dissection before NIR/ICG fluorescence technology activation and sent to the coordinating center. The criteria suggested by Giacopuzzi et al. (37) are used in order to assess the completeness of lymphadenectomy performed with the “naked eye.” These inclusion criteria aim to ensure high-quality standards of surgery.

## Discussion

Despite encouraging results from Eastern countries, the prognosis of gastric cancer in Western population remains poor (1, 2). Both tumor and patient features, such as a more aggressive biological behavior, an advanced stage at diagnosis, an older age, a higher BMI, and the presence of multiple comorbidities, contribute to this dismal prognosis (20).

Surgery remains the best treatment option for patients with resectable gastric cancer (4). Several efforts have been made to safely extend surgical indications beyond traditional limits, such as the introduction of conversion surgery in stage IV gastric cancer as proposed by Yoshida et al. (45), and to improve the quality of surgery by using novel imaging devices. The introduction of NIR/ICG technology in the surgical treatment of AGC has gained growing attention, and several studies suggesting its potential advantage have been published (24, 25). However, several questions regarding NIR/ICG technology application remain unanswered and its actual clinical value is still a matter of debate.

Since ICG allows us to easily distinguish lymphatic vessels and LNs from the surrounding fatty tissue and vascular structures (14), the intraoperative application of NIR/ICG technology might help surgeons to improve lymphadenectomy (21, 25, 27). In the RCT by Chen et al., when residual LNs containing fluorescence were detected in the dissected area, a complementary dissection was performed; moreover, if fluorescent LNs were detected outside the planned dissection area, further dissection beyond D2 lymphadenectomy was performed, configuring *de facto* a D2+ lymphadenectomy. The authors reported a significantly higher mean number of retrieved LNs in the ICG group as compared with the non-ICG group (mean 50.5 vs. 42.0, *p* < 0.001) (27). Of note, current evidence does not support the routine adoption of D2+ lymphadenectomy except for selected cases (4, 9, 46).

On the contrary, Lan et al. reported no significant difference in the number of retrieved LNs among patients who underwent robotic gastrectomy with or without NIR/ICG technology application (23). Although binding to interstitial proteins (albumin, globulins, etc.) and spreading in the lymphatic vessels, ICG has a twofold limit. Firstly, it has a low sensitivity for metastatic LNs (reported 56.3%) and should not therefore be considered a metastatic nodal tracer (27). In addition, it is not specific for LNs because soft tissues without LNs can be occasionally stained with ICG (25).

For these reasons, although lymphadenectomy is one of the main steps in curative-intent surgery for gastric cancer and nodal status is the most important prognostic factor for survival, whether the overall number of harvested LNs should be considered the best indicator of the NIR/ICG technology clinical value remains a matter of debate.

With the present study protocol, we address this issue from a different perspective, evaluating whether the application to NIR/ICG technology can lead to a CSC. Moreover, the incidence of CSC is evaluated in a cohort of patients undergoing surgical intervention with D2 lymphadenectomy performed by expert surgeons in Western hospitals.

## Study Limitations

The iGreenGO Study presents several potential limitations: first of all, the time interval for ICG injection is wide, potentially generating heterogeneity in the methods between participating centers. However, the ideal time for ICG injection is not universally established and a wide time interval is necessary in order to encounter clinical practice and organizational aspects of all participating centers. In addition, in the case of stenotic cancers, ICG injection can be performed at four proximal tumor points. In this case, the injections and the lymph nodes seen may not be representative of all the lymphatic drainage pathways. However, specific subgroup analyses were performed in order to mitigate this possible bias.

Lastly, the sequence for D2 nodal station dissection is not standardized and it is left to the preference of each participating center. However, the pictures taken after lymphadenectomy and the sole inclusion of centers that have performed at least 20 gastrectomies *via* a minimally invasive approach before participating in the study aim to offset these differences. Furthermore, the inclusion of different surgical approaches (laparoscopic and robotic) should increase the reproducibility of the results.

## Conclusion

The iGreenGO Study is the first study to investigate the clinical value of NIR/ICG technology, in terms of intraoperative CSC, in a Western cohort of patients undergoing surgical treatment of AGC. The patterns of ICG fluorescence distribution into the abdominal regions, together with the preoperative clinical variables potentially associated with CSC, are also investigated.

The results from the present study can further clarify the real role of NIR/ICG technology in helping the surgeon during lymphadenectomy for AGC, which is an unanswered question at the present time. Centers interested in participating in the study are invited to contact the corresponding author.

## Data Availability Statement

The raw data supporting the conclusions of this article will be made available by the authors, without undue reservation.

## Ethics Statement

The studies involving human participants were reviewed and approved by Comitato Etico Milano Area 3, Piazza Ospedale Maggiore 3 - 20162 - Milano. The patients/participants provided their written informed consent to participate in this study. Written informed consent was obtained from the individual(s) for the publication of any potentially identifiable images or data included in this article.

## Author Contributions

PL conceptualized and designed the study. GF, MMa, CC, SG, GB, RR, UF, LB, FS, and IG contributed to the study design. VN and SB organized the database. DG performed the statistical analysis presented in the protocol. PL and VN wrote the first draft of the manuscript. MMa, PM, MG, and VN wrote sections of the manuscript. MMu and EF organized the endoscopic setting of the study. All authors contributed to manuscript revision and read and approved the submitted version.

## Conflict of Interest

The authors declare that the research was conducted in the absence of any commercial or financial relationships that could be construed as a potential conflict of interest.

## Publisher’s Note

All claims expressed in this article are solely those of the authors and do not necessarily represent those of their affiliated organizations, or those of the publisher, the editors and the reviewers. Any product that may be evaluated in this article, or claim that may be made by its manufacturer, is not guaranteed or endorsed by the publisher.
